# Extracorporeal Membrane Oxygenation in Infarct-Related Cardiogenic Shock

**DOI:** 10.3390/jcm11051256

**Published:** 2022-02-25

**Authors:** Anne Freund, Steffen Desch, Janine Pöss, Dmitry Sulimov, Marcus Sandri, Nicolas Majunke, Holger Thiele

**Affiliations:** 1 Department of Internal Medicine/Cardiology, Heart Center Leipzig, University of Leipzig, Strümpellstr. 39, 04289 Leipzig, Germany; steffen.desch@medizin.uni-leipzig.de (S.D.); janine.poess@medizin.uni-leipzig.de (J.P.); dmitry.sulimov@medizin.uni-leipzig.de (D.S.); marcus.sandri@medizin.uni-leipzig.de (M.S.); nicolas.majunke@medizin.uni-leipzig.de (N.M.); holger.thiele@medizin.uni-leipzig.de (H.T.); 2 Leipzig Heart Institute, 04289 Leipzig, Germany; 3 German Center for Cardiovascular Research (DZHK), 10785 Berlin, Germany

**Keywords:** acute myocardial infarction, cardiogenic shock, extracorporeal membrane oxygenation, extracorporeal life support

## Abstract

Mortality in infarct-related cardiogenic shock (CS) remains high, reaching 40–50%. In refractory CS, active mechanical circulatory support devices including veno-arterial extracorporeal membrane oxygenation (VA-ECMO) are rapidly evolving. However, supporting evidence of VA-ECMO therapy in infarct-related CS is low. The current review aims to give an overview on the basics of VA-ECMO therapy, current evidence, ongoing trials, patient selection and potential complications.

## 1. Introduction

Cardiogenic shock (CS) is the leading cause of death in hospitalized patients with acute myocardial infarction (AMI) [1]. Up to 10% of AMI patients develop CS, with left ventricular (LV) failure being the leading cause (up to 80% of patients), followed by right ventricular failure and mechanical complications of AMI [2]. Despite major advances in acute cardiac care, mortality remains high, reaching 30–50% during the first 30 days [3,4] To date, revascularization of the culprit lesion is the only causal and effective evidence-based treatment [5,6]. The quest for further improvement of the treatment situation therefore continues and, in particular, the use of active mechanical circulatory support devices is rapidly evolving.

Next to percutaneous LV assist devices, veno-arterial extracorporeal membrane oxygenation (ECMO), also called extracorporeal life support (ECLS), is the major representative of mechanical circulatory support in CS. Compared to other mechanical circulatory support devices, VA-ECMO is able to give full hemodynamic and respiratory support. With 80% of all cases, CS is the leading entity for VA-ECMO use. Particularly between 2010 and 2015, its use increased exponentially [7]. In addition to facilitated availability, new percutaneous techniques for insertion, and the development of smaller and easier to use systems, this period coincides with the time when randomized controlled trials (RCT) showed that the intra-aortic balloon pump (IABP), as a former standard treatment option in AMI-CS, did not provide a survival benefit.

## 2. Basic Operating Principle of VA-ECMO

**Figure 1:**
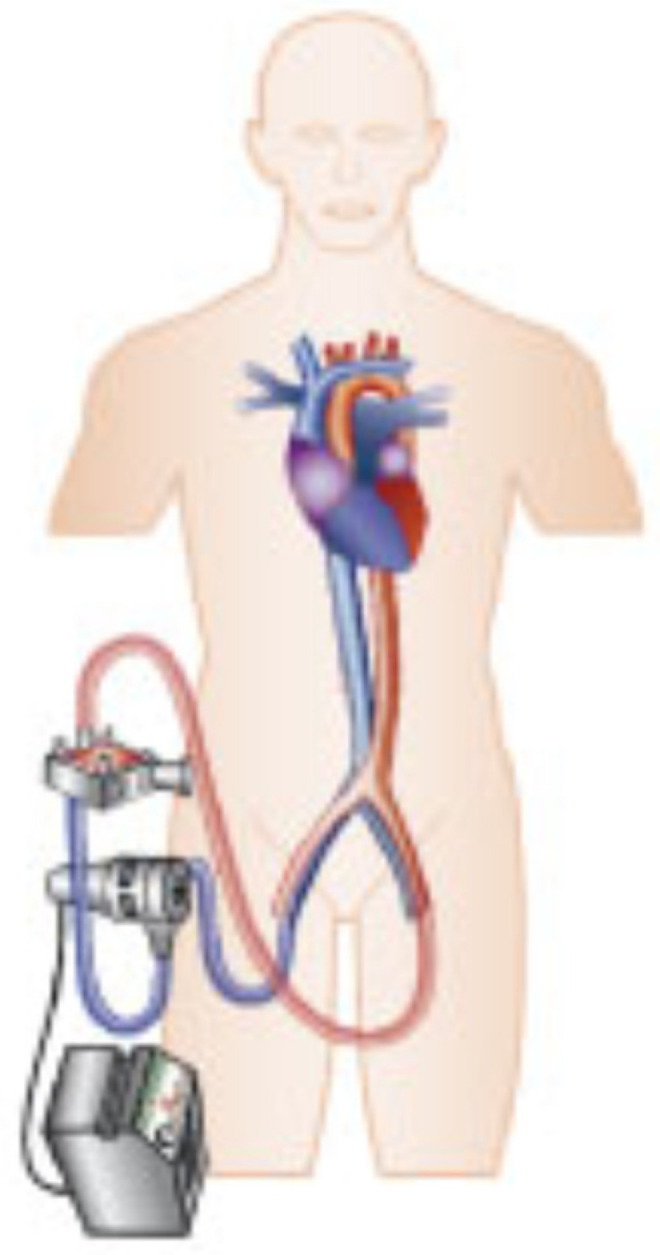
Basic overview VA-ECMO.

The detailed structure of ECMO devices vary between manufacturers. Basically, the VA-ECMO system contains of (1) an inflow cannula transporting blood from a central vein to the pump, (2) a pump with, today almost always, centrifugal flow to keep hemolysis to a minimum, (3) a membrane oxygenator able to fully undertake oxygenation and decarboxylation of the blood, (4) a blood warmer, and (5) an outflow cannula leading to a central artery (Figure 1). The device is, thus, able to give biventricular hemodynamic support. Cannulation can be performed either centrally (via right atrium and aorta or subclavian artery) or peripherally (predominantly via femoral vessels), which is nowadays more frequently chosen in non-post-cardiotomy CS. A major advantage of peripheral access is the less invasive approach and the absent need for a thoracic surgical intervention. This way, experienced centers without on-site cardiac surgery might also perform VA-ECMO therapy. However, in awake patients, central cannulation should be considered to allow for early mobilization. Additionally, severe peripheral vascular disease can be an indication for central cannulation. During VA-ECMO therapy, continuous parenteral anticoagulation is generally considered mandatory to avoid thrombotic complications.

## 3. Evidence of VA-ECMO Therapy in AMI-CS

Despite the steadily increasing use, available evidence of VA-ECMO in AMI-CS is low and guideline recommendations are relatively weak. European heart failure guidelines recommend a short-term percutaneous mechanical circulatory support in selected patients with refractory CS (class of recommendation IIa, level of evidence C) [8]. American guidelines only recommend considering VA-ECMO use in the setting of refractory cardiac arrest [9].

Outcome data on VA-ECMO in AMI-CS are mostly reduced to non-randomized trials. Sattler et al. showed in a very small retrospective single-center study in AMI-CS patients a higher number of survivors with VA-ECMO at short-term follow-up compared to patients without VA-ECMO [10]. In another small retrospective analysis, Sheu et al. demonstrated a survival benefit for patients with AMI and profound CS (defined as systolic blood pressure < 75 mmHg despite medical and IABP treatment) [11]. In a meta-analysis including these two small studies covering only a total of 95 patients, VA-ECMO therapy was associated with improved survival at 30 days (absolute risk difference 0.33, 95% CI 0.14 to 0.52; *p* = 0.0008) [4]. However, these results need to be interpreted with caution because of the high risk of selection bias and an inclusion period during the early phase of VA-ECMO therapy. In the same meta-analysis, two observational studies were analyzed comparing VA-ECMO with TandemHeart or a percutaneous LV assist device (Impella^®^, Abiomed, Danvers, MA, USA). These showed no mortality benefit for VA-ECMO (absolute risk difference −0.03; 95% CI −0.21 to 0.14; *p* = 0.70).

The only available randomized trial on VA-ECMO in AMI-CS available to date included just 42 patients randomized in a 1:1 fashion to VA-ECMO or medical therapy only, in addition to revascularization [12]. The majority of patients experienced cardiopulmonary resuscitation (CPR) before study inclusion. With respect to the primary endpoint of LV ejection fraction, no difference was shown between the two groups at 30 days (50.0% in the VA-ECMO group (IQR: 44.0% to 59.0%) versus 50.8% in the control group (IQR: 47.2% to 60.6%), *p* = 0.86). Furthermore, there was no difference with respect to secondary endpoints such as all-cause mortality (19% in the VA-ECMO group vs. 33% in the control group, *p* = 0.37), stroke or bleeding. When comparing only in-hospital survivors, VA-ECMO therapy was associated with a longer intensive care unit stay and duration of mechanical ventilation. However, considering the comparatively low overall mortality and a median LV ejection fraction of 50% after 30 days, the severity of CS in the included patients may have been only modest. Further limitations include a higher number of diseased coronary vessels in the control group, which is known to be an independent predictor of worse outcome in CS [13,14].

Currently, three large randomized trials (RCT) are evaluating the use of VA-ECMO in AMI-CS (ECLS-SHOCK, EURO-SHOCK and ANCHOR). Detailed characterizations of the three trials are displayed in Table 1. All three trials are powered to assess potential differences in mortality or the combined endpoint of mortality and the requirement for active mechanical circulatory support.

Patients with CS due to mechanical complications of AMI play a special role in VA-ECMO therapy. In these, VA-ECMO might be used as an option for bridging to surgical or interventional therapy, which is often performed after an interval of one week or longer. Again, there is little evidence. In contrast to the European heart failure guidelines, guidelines in acute coronary syndromes recommend short-term mechanical circulatory support with a class of recommendation IIb, level of evidence C for ventricular septal rupture and refractory CS [15]. A recent review addressed the use of active mechanical circulatory support in the setting of ventricular septal defects [16].

## 4. Patient Selection

VA-ECMO therapy is an invasive treatment strategy frequently associated with complications, which can lead to worse outcomes, and is further described in the chapter below. The use of VA-ECMO is by no means a guarantee of favorable outcome. Short-term survival of AMI-CS treated with VA-ECMO reaches 25–45% in registries [11,17].

Patient selection is therefore of high importance until further evidence is available.

Absolute contraindications are rare. In AMI-CS patients with non-corrected aortic dissection, severe aortic regurgitation or uncontrolled bleeding constituting a contraindication for parenteral anticoagulation VA-ECMO should be avoided. VA-ECMO therapy in elderly patients is associated with higher mortality and should therefore be carefully evaluated. A small retrospective analysis of VA-ECMO in AMI-CS showed that mortality reached 80% in patients older than 60 years [18].

In general, VA-ECMO should not be used in cases where acute or chronic conditions make the recovery unlikely, as well as when the therapy cannot lead to a next step in patient care (bridge to recovery, permanent mechanical circulatory support or transplant).

Different scores were developed to assess CS severity and to support future identification of potentially suitable patients for mechanical circulatory support (e.g., IABP-SHOCK II Score, CardShock Score) [19,20]. Since these scores provided only modest predictive discrimination on external validation [21], the decision for mechanical circulatory support in clinical routine should not be based solely on these scores. The more objective CLIP-score based on laboratory values only gave promising results on predicting mortality in a recent publication [22].

Next to general AMI-CS risk models, prediction scores derived from CS cohorts treated with VA-ECMO due to refractory shock were developed [23,24]. With the ENCOURAGE Score, a score is available for VA-ECMO in refractory AMI-CS giving acceptable discrimination between risk groups on first validations [25]. The PREDICT Score incorporates only three point-of-care biomarkers (arterial lactate, pH and standard bicarbonate concentration) and reveals good discrimination with respect to in-hospital mortality in VA-ECMO therapy for CPR or CS irrespective of the timing of the score assessment (one, six or twelve hours) [26]. However, predicting mortality during VA-ECMO therapy is not sufficient to make a decision for or against VA-ECMO initiation as patients also need to be identified who would survive AMI-CS without mechanical circulatory support and might, thus, be harmed as a consequence of potential VA-ECMO complications.

The Society for Cardiovascular Angiography and Intervention (SCAI) introduced an expert consensus classification system not based on a numerical score but on different CS stages mainly depicted by clinical presentation and with a dynamic assessment over time [27]. The possible advantage over other existing scores is the inclusion of the clinical time course. The goal of SCAI was to homogenize the previously heterogeneous definitions of CS in clinical trials. Validation studies revealed heterogeneous results with deviating outcome data in the respective stages [28,29,30,31]. Therefore, an updated version of the SCAI classification with a further refinement of the individual stages was recently published [32]. In addition, a three-axis model was proposed to contextualize the SCAI shock stages in the complexity of CS. Subsequent validations of the updated classification are needed to evaluate future benefit.

However, in the near future, no score will be able to satisfactorily guide clinical decisions. This applies in particular for the prediction of futility, which is gaining increasing importance against the background of ethical and economic considerations.

## 5. Timing of VA-ECMO Initiation

Retrospective analyses recently indicated that the timing of VA-ECMO therapy might influence outcomes. Lee et al. showed in a multicenter registry covering 362 patients with VA-ECMO for refractory CS that all-cause mortality at 30 days was lower in the group of patients with early VA-ECMO initiation compared to those with late VA-ECMO treatment in a weighted Cox model (HR 0.53; 95% CI: 0.28 to 0.99) [33]. Early VA-ECMO initiation was further associated with reduced ECMO weaning failure and all-cause mortality at one year, without significant differences between groups according to safety measurements.

Although early revascularization is the central pillar of AMI-CS treatment, a retrospective single-center study by Choi et al. indicated a superiority of VA-ECMO initiation prior to revascularization: In a total of 147 patients, 50 patients received VA-ECMO prior to revascularization as opposed to 97 patients after revascularization. The primary endpoints of in-hospital mortality, LV assist device implantation and heart transplantation occurred significantly less frequently in the group with VA-ECMO before revascularization (32.0% vs. 49.5%, OR 0.480, 95% CI 0.235–0.982, *p* = 0.045) [34].

However, the evidence regarding the timing of VA-ECMO is still low considering the retrospective design of the studies and a high risk of bias, especially in the study of Choi et al. Of the ongoing RCTs, VA-ECMO-SHOCK intends VA-ECMO insertion prior to PCI and EURO-SHOCK 30 min after revascularization in cases of ongoing refractory CS.

## 6. Intensive Care Treatment

Evidence-based recommendations on intensive care therapy during VA-ECMO treatment are scarce.

### 6.1. Pulmonary Artery Catheter

For optimal hemodynamic assessment and monitoring, the placement of a pulmonary artery catheter (PAC) becomes of reappearing interest after its use in CS has declined to less than 10% [35]. There are observational data showing that PAC placement prior to the initiation of mechanical circulatory support therapy is associated with improved survival [36]. PAC is a valuable tool to assess the individual hemodynamic phenotype and time course of CS. Especially in VA-ECMO, early detection of an increasing pulmonary capillary wedge pressure (PCWP) can help guiding decisions on venting strategies (see below).

### 6.2. Catecholamine Therapy

Higher levels of pharmacological hemodynamic support were associated with higher mortality in observational studies in CS [37]. Therefore, catecholamine therapy in AMI-CS treated with VA-ECMO should be kept to the possible minimum, although randomized data are missing. A subgroup analysis of the SOAP-II trial showed worse outcomes of dopamine treatment compared to norepinephrine in AMI-CS [38]. The single-center RCT DOREMI recently showed no superiority of dobutamine compared to milrinone in CS. However, little is known about the best strategy of catecholamine therapy during VA-ECMO treatment. A retrospective analysis found worse outcomes in patients with epinephrine treatment compared to dobutamine/levosimendan or no inotropic catecholamine during VA-ECMO for CS or extracorporeal CPR (eCPR) but with a high suspicion of existing selection bias [39].

### 6.3. Mechanical Ventilation

The large majority of patients on VA-ECMO are mechanically ventilated due to respiratory insufficiency following AMI-CS. Positive end-expiratory pressure reduces right and left ventricular afterload. Hypoxic pulmonary vasoconstriction might be reduced following better oxygenation. However, invasive ventilation, especially using high pressures, is associated with lung injuries and infections. As VA-ECMO is able to assure sufficient gas exchange, mechanical ventilation should be performed as lung protective as possible during VA-ECMO.

### 6.4. Renal Replacement Therapy

The need for renal replacement therapy (RRT) is frequent in patients with VA-ECMO, with up to 85% of cases with fluid overload being the common reported indication [40,41]. Currently, one RCT is examining if early initiation of continuous RRT is associated with better survival (NCT03549923). Until the results of this are published, RRT will be mostly restricted to conventional indications [42].

## 7. Extracorporeal Cardiopulmonary Resuscitation

eCPR has a separate role in VA-ECMO therapy. The primary goal is to establish systemic perfusion in refractory cardiac arrest to diagnose and possibly treat the underlying cause. The most common cause of out-of-hospital cardiac arrest remains acute myocardial infarction [43]. Thus, an eCPR approach appears appealing to enable coronary revascularization and restore ventricular function. However, due to the often poor (especially neurologic) prognosis in patients with refractory cardiac arrest, the indication for eCPR should be well-considered, including only patients with characteristics associated with a favorable outcome, e.g., minimal no-flow time, initial shockable rhythm, limited time from cardiac arrest to intervention and others. Smaller observational studies with propensity matching of eCPR and conventional CPR found better survival rates for eCPR (Chen et al.: HR 0.53 for one-year mortality, 95% CI 0.33–0.83, *p* = 0.006; Shin et al.: OR for mortality or significant neurologic deficit at discharge, 0.17, 95% CI 0.04–0.68; *p* = 0.012) in in-hospital cardiac arrest [44,45]. However, out-of-hospital cardiac arrest (OHCA) observational studies in particular, showed conflicting results with respect to survival and neurological outcome. A former systematic review of Holmberg et al. concluded that a meta-analysis of the available data is not possible due to a critical risk of bias and heterogeneity [46]. Afterwards, the first RCT in ECLS was published, including 30 patients with refractory ventricular fibrillation [47]. The trial was terminated early because of posterior probability of ECMO superiority. The primary outcome was survival to hospital discharge, reached in 43% in the eCPR group and in 7% in the control group without ECLS (risk difference 36.2%, 95% credible interval 3.7 to 59.2). In the recently presented Prague-OHCA trial, a total of 264 patients with refractory OHCA were randomized pre-clinically to either a hyperinvasive approach (mechanical compression device, intranasal cooling, admission to catheterization laboratory and evaluation for ECLS) versus standard of care following current guidelines. First analyses showed a potential benefit of the hyperinvasive approach [48]. Several further randomized trials in the eCPR setting are currently being conducted (intra-hospital implementation of VA-ECMO: INCEPTION [NCT03101787], ECPB4OHCA [NCT01605409], intra-hospital implementation of VA-ECMO: ON-SCENE [NCT04620070]).

## 8. Complications of VA-ECMO Therapy

While full biventricular and respiratory support are appealing for bridge to recovery or device/transplant, complications rates of VA-ECMO are still high.

The most frequent include:

- Bleeding
- Clotting
- Hemolysis
- Limb ischemia
- Inadequate LV unloading
- Harlequin syndrome
- Infection

### 8.1. Bleeding/Clotting

Bleeding and thrombotic complications are closely related, and hemostatic balance is a major challenge of VA-ECMO treatment. VA-ECMO therapy requires continuous anticoagulation. There are no specific guidelines on anticoagulation regimens due to a lack of evidence. Usually, unfractionated heparin is used; alternatively, low molecular weight heparins or argatroban. With unfractionated heparin, an activated partial thromboplastin time of 1.5 times above the upper limit is often aimed for. Anticoagulation is necessary to prevent thrombus formation in the oxygenator and subsequent thromboembolic complications to the large foreign surface, which can have disastrous consequences due to the involvement of the central venous and arterial system. Relevant clot formation in the VA-ECMO circuit is described in about 10% of cases, thrombotic stroke less frequently with 4–7% of VA-ECMO cases [7].

Anticoagulation, in turn, increases the risk of bleeding, which is already elevated as a consequence of the consumption of clotting factors because of the large synthetic surface, endothelial injury and shear stress in the extracorporeal circuit. The usually necessary administration of transfusion of blood components leads to immunological responses, which can further increase the bleeding tendency [49]. With newer, coated ECMO circuits, prophylactic anticoagulation regimens might be considered if bleeding risk is high [50]. Bleedings are often located on cannula insertion sites due to the necessary sheath sizes but are not restricted to those. In large registries, hemorrhagic complications are described in up to 44% of VA-ECMO patients, with approximately 2% suffering intracranial hemorrhage [51,52]. Post hoc analyses of the CULPRIT-SHOCK study showed that ECMO therapy was independently associated with an increased incidence of bleeding (OR 3.31; 95% CI 1.64–6.71) [53]. Bleeding, in turn, was an independent predictor of increased 30-day mortality (HR 2.11, 95% CI 1.63 to 2.75; *p* < 0.0001).

### 8.2. Hemolysis

Hemolysis in ECMO patients is common due to an increased shear stress in the circuit. In most cases, the manifestation is low level, especially since the wide-spread use of centrifugal pumps. However, in cases of risk factors, such as the insufficient flow in the outflow cannula leading to a rise in the negative pump head pressure, necessity of a high blood flow, or development of pump head thrombosis, severe hemolysis can occur and is reported in up to 5% of patients [54]. Lyu et al. retrospectively observed, in a total of 84 VA-ECMO patients, that increased levels of plasma free Hb are associated with acute renal failure [55]. Omar et al. even identified severe hemolysis (peak plasma free Hb > 500 mg/L) as an independent predictor of worse outcome [56], although data in this field remain controversial and further studies are needed.

### 8.3. Limb Ischemia

Limb ischemia occurs almost exclusively in patients with peripheral VA-ECMO cannulation in about 3.6% of patients [54]. Therefore, positioning of an additional antegrade sheath allowing for distal limb perfusion should be performed in all peripheral VA-ECMO cases. Initial ultrasound-guided cannulation provides further reduction in access-related complications. It should be kept in mind that a small, non-perfused space forms between the large bore arterial cannula and the antegrade sheath, which can promote thrombus formation leading to possible thromboembolic complications during VA-ECMO removal. Recently, a technique to flush this non-perfused space during removal procedure was introduced to further reduce the rate of limb ischemia [57].

### 8.4. Inadequate LV Unloading

Ventricular unloading under VA-ECMO therapy has come more and more into focus in recent years. Findings suggest that due to the return of blood to the aorta, the afterload of the LV might increase [58]. This may result in a further reduced LV stroke volume leading to an increase in end-diastolic LV pressure, LV distension, pulmonary edema and LV clot formation as a result of inadequate opening of the aortic valve. The largest meta-analysis, so far, including 3930 patients from 16 studies (AMI-CS and post-cardiotomy shock), showed a benefit regarding in-hospital mortality for any venting strategy (OR 0.54; 95% CI 0.42–0.70; *p* < 0.001) [59].

**Figure 2:**
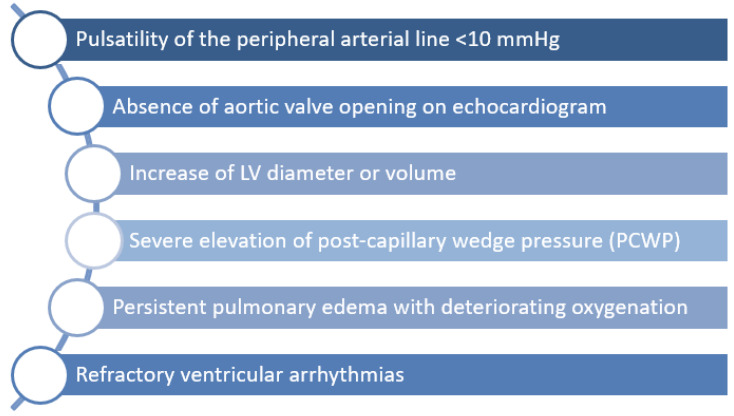
Potential indications for LV venting during VA-ECMO therapy. LV = left ventricular.

Different techniques for venting of the LV have been established. These should be considered in situations outlined in Figure 2 until further evidence is available.

#### 8.4.1. LV Unloading Using LV Microaxial Flow Pump

The so called ‘ECMELLA’ (or ‘ECPELLA’) strategy describes a situation where VA-ECMO is combined with an LV microaxial flow pump (Impella^®^, Abiomed, Danvers, MA, USA). The pump is placed through a femoral or axillary artery and provides unloading by pumping blood from the LV into the ascending aorta.

Retrospective studies showed a survival benefit in patients with this venting strategy compared to patients with VA-ECMO only. The largest propensity-matched study of Schrage et al., analyzed a total of 510 patients from four multinational tertiary care centers and showed an association with lower 30-day mortality of ECMELLA compared to VA-ECMO alone (HR 0.79; 95% CI 0.63–0.98; *p* = 0.03) despite a higher rate of severe bleeding (38.4% in the ECMELLA group vs. 17.9% in VA-ECMO alone), hemolysis (33.6% vs. 22.4%), interventions due to access site-related ischemia (21.6% vs. 12.3%), abdominal compartment syndrome (9.4% vs. 3.7%) and the need for renal replacement therapy (58.5% vs. 39.1%) [60].

#### 8.4.2. LV Unloading Using Intra-Aortic Balloon Counterpulsation

In animal and human in vivo studies, the use of intra-aortic balloon counterpulsation (IABP) showed a significant decreased LV afterload and PCWP [61]. A recent non-randomized analysis showed a beneficial short-term survival in the case of concomitant IABP therapy in VA-ECMO patients, although additional IABP was associated with a higher risk of major bleeding [62] However, in this study the number of patients without IABP was low and selection bias cannot be excluded. A meta-analysis including 14 previous retrospective studies with IABP as an unloading strategy during VA-ECMO therapy showed a reduction in in-hospital mortality for patients with concomitant IABP treatment (OR = 0.61; 95% CI 0.46–0.81; *p* < 0.001), although a higher reduction in mortality for preload targeting venting strategies was displayed [59].

#### 8.4.3. Other Venting Strategies

Further strategies to enhance LV unloading include a pigtail catheter from the LV to the venous ECMO cannula and surgical cannulation of the LV through the apex or percutaneous balloon atrial septostomy. However, these are rarely used in clinical routine. Recent animal data could confirm the advantage of atrial septostomy on hemodynamics [63].

To date, no RCTs of LV unloading in VA-ECMO therapy are available to support a selective or routine use. Currently, two RCTs are being conducted: REVERSE (NCT03431467) aims to randomize a total of 96 patients to either VA-ECMO alone or VA-ECMO with early Impella CP^®^ venting. EARLY-UNLOAD (NCT04775472) is recruiting 116 patients to compare early left atrial septostomy with selective septostomy.

### 8.5. Harlequin Syndrome (North-South Syndrome)

The Harlequin syndrome describes a condition in which, in the event of severely restricted gas exchange in the lungs, insufficiently oxygenated blood enters the coronaries and the upper half of the body via the first branches of the aorta. It occurs exclusively in cases of retrograde aortic blood return (e.g., femoral cannulation). As a consequence of this watershed phenomenon, the risk of myocardial and cerebral hypoxia is increased, especially when the LV starts recovering. Close clinical monitoring and blood samples from the right radial artery are imperative. In the case of suspected Harlequin syndrome, mechanical ventilation should be optimized and the setting of VA-ECMO cannulation should be adapted in case of ongoing differential hypoxia by the implementation of VAV-ECMO or central aortic cannulation to provide antegrade flow [64].

**Figure 3:**
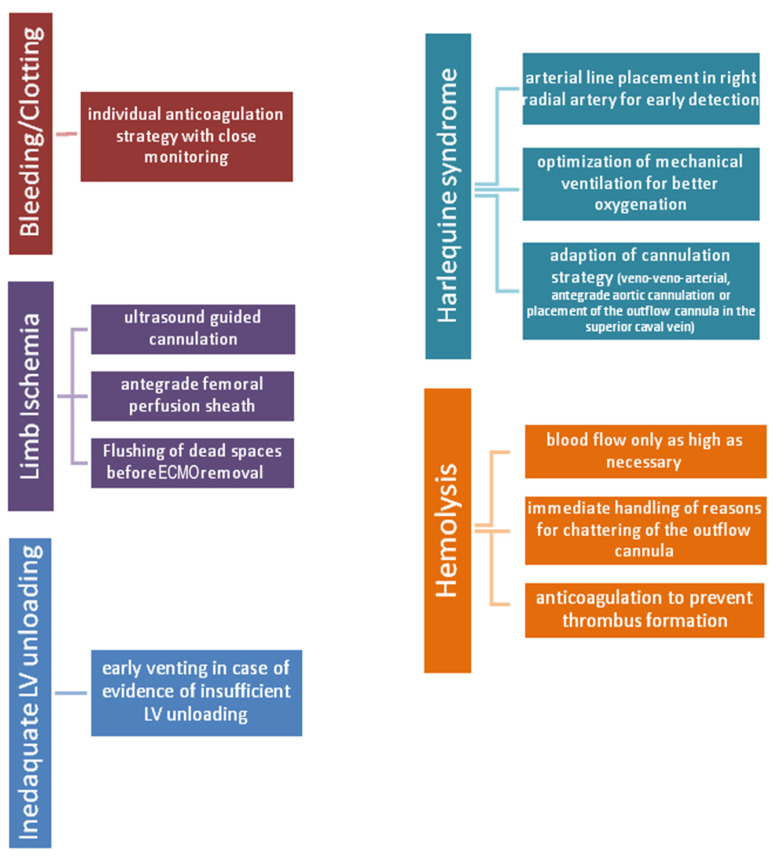
Strategies to avoid potential complications of VA-ECMO therapy. ECMO = extracorporeal membrane oxygenation; LV = left ventricular.

Figure 3 displays an overview on common VA-ECMO complications and possible avoidance strategies.

## 9. Weaning and Decannulation

Once hemodynamic and rhythmic stabilization has been achieved, gradual weaning of VA-ECMO should be considered.

Prior to this, euvolemia should be achieved and echocardiography performed to ensure that the patient’s own cardiac output is adequate. In a small retrospective analysis covering a total of 51 patients with different causes of CS, higher arterial systolic and pulse pressures, aortic VTI (≥10 cm), LVEF (<20–25%), and lateral mitral annulus peak systolic velocity (TDSa, ≥6 cm/s) were found to be positively predictive of successful weaning [65]. Further assessment criteria for ECMO weaning include an improvement in end organ failure and meeting of the therapeutic anticoagulation goal as the risk of thrombus formation increases with the reduction in blood flow [66]. A small retrospective study also showed an increased VA-ECMO weaning success in the case of pre-treatment with levosimendan in post-cardiotomy patients [67]. In contrast, Guilherme et al. showed, in a larger prospective cohort, no superiority of levosimendan treatment [68]. Currently, the French RCT LEVOECMO is recruiting 206 patients with acute CS of different entities comparing levosimendan and a placebo.

Liberation from VA-ECMO can be performed either by a stepwise flow reduction (e.g., 0.5 L steps to a minimum flow of 0.5–1.5 L), or, alternatively, a liberation trial can be implemented with flow reduction in three steps (to 50% of the starting flow, 25% of the starting flow and minimum flow). After 30 min of the respective step, predefined hemodynamic, respiratory, catecholamine and lactate targets should be assessed to decide for continuation or discontinuation of the trial [66].

After weaning, decannulation will be performed depending on the initial cannulation strategy: In the case of central cannulation, surgical removal is obligatory. In the case of peripheral cannulation, different options to achieve hemostasis are available. As described above, attention should be paid to the developing dead-space between the arterial cannula and the antegrade sheath during decannulation [57]. The former standard was placement of a femoral compression system during decannulation. However, in the case of failed hemostasis, bail-out options are limited. Today, vascular closure devices for the arterial access site are often introduced during VA-ECMO removal, such as suture-mediated or collagen-mediated devices. In the case of insufficient hemostasis, multiple devices might be used. Closure devices can also be inserted during the initial cannulation to prepare for later decannulation. However, infectious complications might be increased. Currently, no evidence is available on the best peripheral decannulation strategy.

## 10. Conclusions

The use of VA-ECMO as the only mechanical circulatory support device enabling full biventricular support is steadily evolving. While the concept is appealing in refractory or severe CS, and also as an eCPR strategy, evidence of its use is mainly restricted to relatively small, non-randomized studies, one RCT in CS and two RCT in eCPR. Stronger evidence on patient selection and treatment modalities is urgently needed, especially against the background of a high complication rate.

## Figures and Tables

**Table 1:** Ongoing Randomized Trials of VA-ECMO in AMI-CS.

	ECLS-SHOCK	EURO-SHOCK	ANCHOR
Identifier	NCT03637205	NCT03813134	NCT04184635
Sample Size	420 patients	428 patients	400 patients
First Patient in	June 2019	January 2020	October 2021
Patient enrolment as of January 2022	300	33	<10
Main Inclusion Criteria	Infarct-related CS (STEMI or NSTEMI) < 12 h Arterial lactate > 3 mmol/L Planned revascularization Age: 18–80 years In case of prior CPR: duration < 45 min	Infarct-related CS (STEMI or NSTEMI) Presentation ≤ 24 h after ACS symptom onset Persistence of CGS 30 min after revascularization attempt of culprit coronary artery Arterial lactate > 2 mmol/L Age: 18–90 years	Infarct-related CS (STEMI or NSTEMI) < 24 h) PCI performed or planned in the following 60 min Age >18 years In case of prior CPR: duration < 30 min
Treatment Arms	Optimal medical therapy vs. VA-ECMO plus optimal medical therapy	Optimal medical therapy vs. Early VA-ECMO plus optimal medical therapy	Optimal medical therapy vs. Early VA-ECMO and IABP plus optimal medical therapy
Primary Outcome	All-cause 30-day mortality	All-cause 30-day mortality	Treatment failure at day 30 (death in the ECMO group and death or rescue ECMO in the control group)
Special Characteristics	VA-ECMO arm: VA-ECMO insertion preferably prior PCI Non-VA-ECMO arm: Use of other mechanical circulatory support than VA-ECMO possible in case of defined escalation criteria	VA-ECMO arm: VA-ECMO insertion 30 min until 6 h after PCI Non-VA-ECMO arm: IABP insertion not permitted	VA-ECMO arm: VA-ECMO insertion as soon as possible Non-VA-ECMO arm: Use of IABP not recommended, other mechanical circulatory support devices not permitted

IABP = intra-aortic balloon counterpulsation; (N)STEMI = (non-)ST-segment elevation myocardial infarction; PCI = percutaneous coronary intervention; VA-ECMO = veno-arterial extracorporeal membrane oxygenation.

## Data Availability

Not applicable.
